# Kinetic analysis of the growth rate of sporadic and hereditary medullary thyroid carcinoma: comparing the postoperative calcitonin-doubling rate with the hypothetical preoperative tumor volume-doubling rate

**DOI:** 10.1186/s13044-020-00087-6

**Published:** 2020-07-21

**Authors:** Minoru Kihara, Akira Miyauchi, Hiroo Masuoka, Takuya Higashiyama, Yasuhiro Ito, Akihiro Miya

**Affiliations:** Department of Surgery, Kuma Hospital, 8-2-35 Shimoyamate-dori, Chuo-ku, Kobe, Hyogo 650-0011 Japan

**Keywords:** Kinetic analysis of the growth rate, Doubling rate, Medullary thyroid carcinoma, Calcitonin, Tumor volume

## Abstract

**Background:**

Our previous kinetic analyses of changes in the tumor volume (TV) of papillary thyroid microcarcinomas during active surveillance revealed that the tumors’ growth varied over time from rather rapid growth to shrinkage and that the hypothetical TV-doubling rates (DRs) before the patients’ presentation were much larger than their observed TV-DRs, indicating that rapid growth phases preceded their presentation. Whether this phenomenon also occurs in medullary thyroid carcinoma (MTC) was unknown.

**Methods:**

We retrospectively analyzed the cases of 46 MTC patients (18 hereditary, 28 sporadic; 9–80 years old at surgery, median 53.5 years; 19 males and 27 females) with elevated postoperative calcitonin (Ct) measured with the electrochemiluminescence immunoassay suggesting persistent disease. We calculated each patient’s Ct-DR and his/her hypothetical TV-DR, using the tumor size and age at surgery.

**Results:**

Ct-DRs (/year) after surgery were > 0.5, 0.1–0.5, − 0.1–0.1, and < − 0.1 in 9, 21, 12, and 4 patients, respectively (median 0.17). The hypothetical TV-DRs (/year) before surgery were > 1, 0.5–1.0, 0.1–0.5 and < 0.1 in 11, 21, 14, and 0 patients, respectively (median 0.60). The hypothetical TV-DR was higher than the observed Ct-DR in 41 of the 46 MTC patients and all 18 patients with hereditary MTC, suggesting that a rapid growth phase preceded surgery in these patients.

**Conclusions:**

In this series of MTC patients, the pre-surgery calculated hypothetical TV-DRs were significantly higher than the Ct-DRs observed post-surgery, suggesting that there were rapid growth periods before surgery in the vast majority of these MTC patients.

## Introduction

Medullary thyroid carcinoma (MTC) is an uncommon malignant tumor derived from the C cells (calcitonin-producing cells) of the thyroid gland, and it presents in a sporadic or hereditary variant. Serum calcitonin (Ct) is a very sensitive and specific tumor marker for MTC. In 1984, Miyauchi et al. found that in patients with MTC who had elevated serum Ct values postoperatively, the serum Ct values changed exponentially; they reported that the Ct-doubling time (Ct-DT) was a strong prognostic factor for MTC [1]. Twenty-one years later, Barbet et al. performed multivariate analyses on possible prognostic factors for MTC and found that among various clinicopathological factors, the Ct-DT remained an independent prognostic factor [2]. Some of their patients showed a decrease in their serum Ct values over time, rendering negative values for their Ct-DTs. This created a discontinuity problem among positive and negative Ct-DT values. Barbet et al. were very smart in solving this problem: They converted the Ct-DT to 1/Ct-DT for statistical analyses. We recently encountered a similar problem in our kinetic study of tumor volumes during the active surveillance of patients with low-risk papillary thyroid microcarcinomas, since some of these patients also showed decreases in their tumor volumes over time. We converted the tumor volume-doubling time (TV-DT) to its inverse (i.e., 1/TV-DT), and we proposed that this value be called the ‘doubling rate (DR)’, because it is designed to indicate the number of doublings (or halvings) that occur per unit of time [3]. With the TV-DR we were able to determine the tumor volume kinetics during active surveillance in individual patients in a single figure [3].

For the investigation of tumors’ growth before the patients’ clinical presentation, we calculated the hypothetical TV-DR — which is an estimate of the tumor growth rate before presentation — by using the patient’s age and the tumor size at presentation, assuming that a single 10-μm cancer cell was present at the patient’s birth and grew at a constant rate. If a tumor arose later than the birth time point, the tumor’s growth rate should be higher than this estimate. If the tumor’s growth was not constant and there was a slow-growth phase, there should be a period of more rapid growth for the cancer cell to grow to the size observed at presentation. Therefore, the hypothetical TV-DR is the lowest tumor growth rate before presentation.

Our research revealed that in most of the patients, the hypothetical TV-DR was much larger than the TV-DR observed during active surveillance, suggesting that the tumors’ growth before the patients’ presentation had been much more rapid. In other words, the papillary microcarcinomas had undergone a spontaneous decrease in their tumor growth rates. Some of the tumors even shrank after their presentation. Whether a similar phenomenon also occurs in MTCs has been unknown, and we conducted the present study to investigate the natural history of MTCs before presentation by comparing the Ct-DRs observed after surgery with the hypothetical TV-DRs that were estimated before surgery.

## Materials and methods

### Subjects

From January 2005 to December 2018, 199 patients underwent their initial surgery for MTC at our hospital. Of these, 46 patients (23%) had postoperative Ct values ≥10 pg/ml measured with the electrochemiluminescence immunoassay, suggesting biochemically persistent disease, and they were enrolled in this study (Table 1). They were 19 males and 27 females, ages 9–80 years old at surgery (median 53.5 years). The maximum diameter of the patients’ largest tumors ranged from 5 mm to 75 mm (median 20 mm). All patients underwent a germline *RET* mutation analysis preoperatively. Twenty-eight patients had no *RET* gene mutations. The other 18 patients had the *RET* gene mutations that are regarded as posing the highest risk (*n* = 3 patients), high risk (*n* = 7) and moderate risk (*n* = 8) according to the newest American Thyroid Association (ATA) guidelines [4]. None of the patients underwent any additional surgery, tyrosine-kinase inhibitor therapy, or external beam radiotherapy after the initial surgery. None of the patients had hyperparathyroidism, hepatic cirrhosis, or renal insufficiency. None were treated with a drug that increases calcitonin secretion (e.g., omeprazole, beta-blockers, and glucocorticoids).

**Table 1:** Clinicopathological characteristics in hereditary MTC patients and in sporadic MTC patients

Variable	Total	Sporadic MTC	Hereditary MTC	*p*-value
No. of patients	46	28	18	
Age at surgery, yrs. ^a^	53 (9–80)	59 (25–80)	28 (9–59)	< 0.001
Sex, no. (%)				
Male	19	14	5	0.220
Female	27	14	13	
Max. tumor size at surgery, mm^a^	20 (5–75)	20 (9–63)	21 (5–75)	0.991
Extrathyroid extension:^b^				
Yes	2	1	1	0.999
No	46	27	17	
Ki67 labeling index:				
< 5%	39	25	14	0.163
5–10%	2	0	2	
> 10%	4	3	1	
Unknown	1	0	1	
pN (8th UICC/AJCC staging system):				
pN0	3	1	2	0.594
pN1a	3	2	1	
pN1b	40	25	15	
Stage (8th UICC/AJCC staging system):				
I	3	1	2	0.594
III	3	2	1	
IVA	40	25	15	
Ct-DR, per year:^a^	0.17 (−1.25–5.70)	0.17 (−0.42–5.70)	0.21 (−1.25–1.90)	0.753
hTV-DR, per yr ^a^	0.60 (0.26–3.65)	0.50 (0.26–1.10)	1.21 (0.50–3.65)	< 0.001
Observation period, yrs	4.6 (1.0–4.6)	4.6 (1.0–4.6)	4.6 (1.0–4.6)	0.079
No. of Ct measurements	9 (4–10)	7 (5–10)	9 (4–9)	0.018

*Ct-DR* calcitonin doubling rate, *hTV-DR* hypothetical tumor-volume doubling rate, *MTC* medullary thyroid carcinoma

^a^Medians are in parentheses. ^b^Significant extension based on intraoperative findings

### Calcitonin assay

In Japan, calcitonin was measured before April 2015 by using the solid two-site immunoradiometric assay. After that point, calcitonin has been measured by an electrochemiluminescence immunoassay (ECLIA). In the present study, we used only the Ct values measured with an ECLIA. The patients’ Ct values were measured by a laboratory (SRL Co., Tokyo) using the Elecsys® Calcitonin test system (Roche Diagnostics, Tokyo). According to the manufacturer, the normal ranges of basal serum calcitonin are ≤9.52 pg/mL for males and ≤ 6.40 pg/mL for females, with a lower detection limit of quantification of 0.5 pg/mL.

### Measurement of serum Ct and calculation of the Ct-DR

The patients’ post-operative serum Ct was measured two to four times per year. A median number of total Ct measurements was 9, ranging from 4 to 10 in this study (Table 1). We calculated each patient’s Ct-DR as described [3]. This calculation is easily performed using the Doubling Time, Doubling Rate & Progression Calculator (freely available from: http://www.kuma-h.or.jp/english/).

### Hypothetical tumor-volume doubling rate (hTV-DR)

We calculated the hypothetical (h)TV-DRs by using the tumor size and the patient’s age at surgery, assuming that a single 10-μm cancer cell was present at the patient’s birth and grew at a constant rate [3]. Tumor diameters were measured with the use of ultrasonography shortly before the patients’ surgeries. The maximum diameter (D1) and the diameter in the direction perpendicular to the maximum diameter (D2) were measured. The tumor depth value was often not reliable because of ultrasound shadowing. The volume of each tumor at surgery was calculated using the ellipsoid equation: π/6 × D1 × D2^2^ [5]. ‘Time’ is the time interval between the patient’s birth and surgery, i.e., the age (years) at surgery. The hTV-DR was calculated using the Doubling Time, Doubling Rate & Progression Calculator.

### Statistical analysis

The statistical tests used to compare the differences between the groups were the chi-square test for independence, the Mann-Whitney U-test for skewed variables, and the Wilcoxon signed-rank test for paired skewed variables. A *p*-value < 0.05 was regarded as significant. All analyses were performed using StatFlex 6.0 software (Artech, Osaka, Japan).

## Results

Table 1 summarizes the results of our comparison of the demographic and clinicopathological features of the patients with sporadic and hereditary MTC. The median age at surgery of the patients with hereditary MTC was significantly lower than the ages of the patients with sporadic MTC (*p* < 0.001). There was no significant difference in sex, max. Tumor size, extrathyroid extension, Ki67 labeling index, pN, or TNM stage between the sporadic MTC patients and the hereditary MTC patients, both groups having biochemically persistent disease postoperatively.

**Fig. 1:**
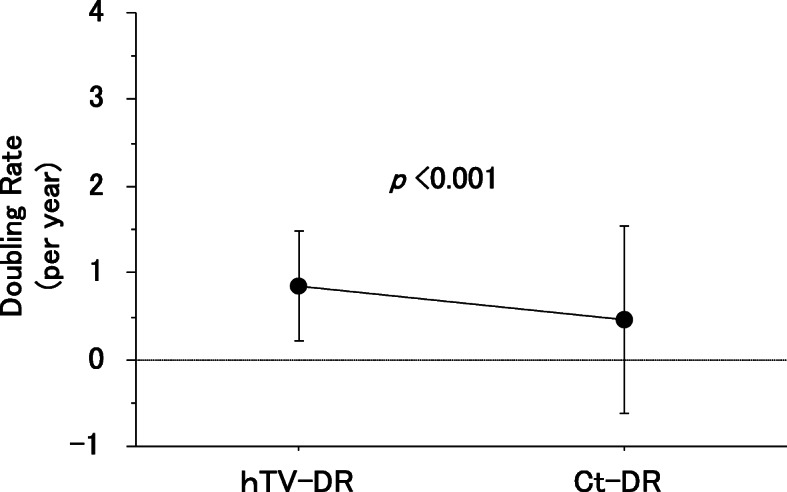
The calcitonin (Ct)-DR and the hypothetical tumor-volume doubling rate (hTV-DR) values of the 46 MTC patients

The Ct-DRs (/year) after surgery in the 46 patients ranged from − 1.25 to 5.70 with a median of 0.17. The hTV-DR values (/year) before surgery ranged from 0.26 to 3.65 with a median of 0.60. The hTV-DR values were significantly higher than the Ct-DR values (p < 0.001), suggesting that the tumor growth in these patients was more rapid before surgery (Table 1, Fig. 1).

We divided the patients’ Ct-DR values into four categories: rather rapid growth, slow growth, stable, and decrease, using cutoffs at 0.5, 0.1, and − 0.1 per year. In the 46 patients, the Ct-DR (/year) was > 0.5, 0.1–0.5, − 0.1–0.1, and < − 0.1 for 9, 21, 12, and 4 patients, respectively (Table 2). The patients with sporadic MTC and those with hereditary MTC showed similar distributions of Ct-DR values (*p* = 0.923). Although these 46 patients had biochemically persistent disease, 26% of them showed stable disease and 9% of them showed a decrease in serum Ct values that may indicate shrinkage of their tumors. By definition, the hTV-DR should be larger than 0. The hTV-DR (/year) values were > 1, 0.5–0.1, 0.1–0.5, and < 0.1 in 11, 21, 14, and 0 patients, respectively (Table 3). The 18 patients with hereditary MTC had significantly higher hTV-DR values compared to the 28 patients with sporadic MTC (*p* < 0.001), possibly due to older age in sporadic MTC.

**Table 2:**
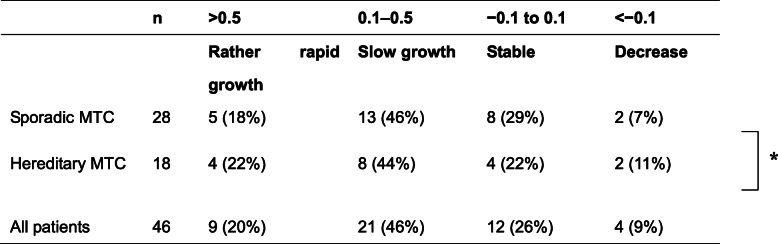
Calcitonin doubling rates in 28 patient with sporadic MTC and 18 patients with hereditary MTC

*p = 0.923. MTC; medullary thyroid carcinoma

**Table 3:** Hypothetical tumor-volume doubling rates in 28 patient with sporadic MTC and 18 patients with hereditary MTC

	n	> 1	0.5–1.0	0.1–0.5	< 0.1
		Rapid growth	Rather rapid growth	Slow growth	Very slow
Sporadic MTC	28	1 (4%)	13 (46%)	14 (50%)	0 (0%)
Hereditary MTC	18	10 (56%)	8 (44%)	0 (0%)	0 (0%)
All patients	46	11 (24%)	21 (46%)	14 (30%)	0 (0%)

**Fig. 2:**
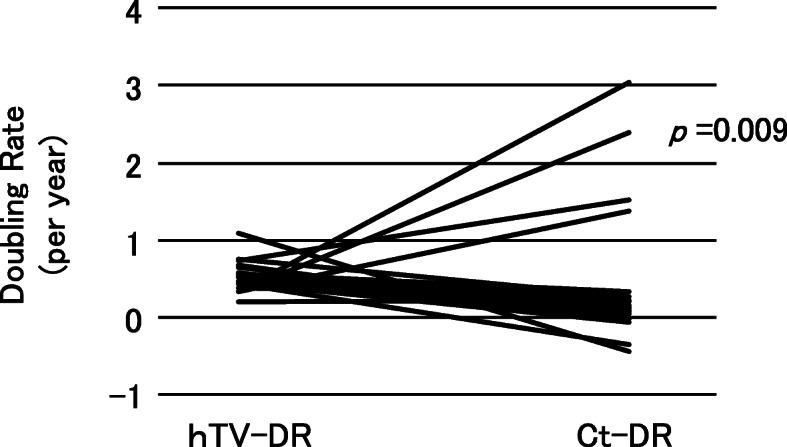
The Ct-DR and hTV-DR values of the 28 sporadic MTC patients

**Fig. 3:**
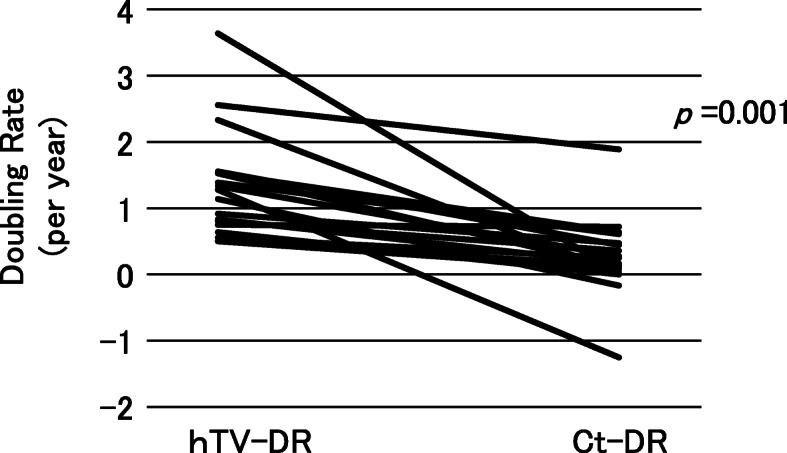
The Ct-DR and hTV-DR values of the 18 hereditary MTC patients

In the 28 patients with sporadic MTC, their Ct-DR values (/year) (median 0.17, range − 0.42–5.70) were significantly lower than their hTV-DR values (/year) (median 0.50, range 0.26–1.10) (*p* = 0.009) (Fig. 2). However, the Ct-DR values of four patients were higher than their hTV-DR values, suggesting that the preoperative tumor growth was slower than the postoperative growth, or the tumor arose much later than at birth in these patients. On the other hand, in all 18 of the patients with hereditary MTC, the Ct-DR values (/year) (median 0.21, range − 1.25–1.90) were significantly lower than the hTV-DR values (/year) (median 1.21, range 0.50–3.65) (*p* = 0.001), suggesting there was a more rapid tumor growth phase before surgery in all of these patients (Fig. 3).

## Discussion

Collins et al. [6] reported that the growth of human tumors was exponential, and they proposed that the tumor growth rate is best described as tumor DT. Other research concerning the changes in serum Ct and carcinoembryonic antigen (CEA) values over time in patients with MTC who had persistent disease postoperatively and studies of the serum thyroglobulin values in patients with papillary thyroid carcinoma (PTC) after a total thyroidectomy has demonstrated that these values changed exponentially and that the DTs of these tumor marker values were very strong prognostic factors, supporting the concept described by Collins et al. [1, 7]. Thus, the DT is a well-validated parameter for the analyses and expressions of tumor growth.

However, the DT has two major limitations. First, if the volume of some of the tumors decreases over time, the tumors’ DTs become negative values, which creates a discontinuity problem among positive and negative DT values. Second, the magnitude of the DT values is the opposite of the magnitude of the tumor growth rate. However, if the inverse of the DT is used (i.e., 1/DT), these limitations are resolved, and these inverse values should indicate the number of doublings that occur per unit of time. We thus proposed calling the 1/DT value the ‘doubling rate’ [3].

With the TV-DR we were able to demonstrate tumor volume kinetics of papillary microcarcinomas during active surveillance in individual patients [3]. Very interestingly, the volume of 17% of the tumors in that study decreased over time, showing negative TV-DRs values. Our calculation of the hypothetical TV-DR (an estimate of the lowest tumor growth rate) before presentation using the patient’s age and tumor size at presentation showed that this value was much higher than the TV-DR observed during active surveillance in most of the patients, suggesting that the tumor growth before presentation had been much more rapid [3]. We also observed that in patients with papillary thyroid microcarcinoma during active surveillance, the probability of tumor progression greatly decreased with age [8, 9]. These data indicated that the spontaneous deceleration of tumor growth with age is a rather common phenomenon in papillary thyroid microcarcinomas.

To determine whether a similar phenomenon occurs in medullary thyroid carcinoma, we calculated the hTV-DR applying our above-described hypothesis, and we also calculated the Ct-DR in the 46 patients with MTC showing biochemically persistent disease postoperatively. The estimated hTV-DRs were significantly higher than the observed Ct-DRs. This difference was more pronounced in the 18 patients with hereditary MTC than in the 28 patients with sporadic MTC. This thought experiment suggests that a rapid growth phase preceded surgery in the vast majority of the present patients with MTC, especially in the 18 patients with hereditary MTC. Even in patients with sporadic MTC whose hTV-DRs were lower than those of hereditary MTC, possibly due to higher age of the former patients, their hTV-DRs were often higher than their Ct-DRs. These data thus suggest that most of the MTCs grew more rapidly before surgery. In other words, the growth rate of most of the MTCs was lower during follow-up than hypothetical maximal value during early stages of tumor development; the reason(s) for this deceleration remain to be clarified. This was especially evident for the hereditary MTCs. Hereditary MTCs arise at younger ages than sporadic MTCs [10–13], yet their prognosis is not worse than that of sporadic MTCs [13–15]. Four of the presented 28 patients with sporadic MTC had Ct-DR values that were higher than their hTV-DR values. This may be due to too small estimates of hTV-DR in these patients since they had large age values (old ages) at surgery. The actual origin of the tumor should have been much later than birth in these patients. Alternatively, the growth rate in these patients became more rapid after surgery.

Kasahara et al. reported a spontaneous decrease in serum thyroglobulin levels in pediatric patients with papillary thyroid carcinoma after total thyroidectomy [16]. We reported a spontaneous postoperative deceleration in the Ct-DT of patients with persistent hypercalcitoninemia in a long-term follow-up [17]. Ito et al. reported that the TV-DR values of the majority of papillary thyroid microcarcinomas decreased during the follow-up, after they showed increases in their tumor size during active surveillance [18]. These data suggest that a spontaneous deceleration of tumor growth following presentation or surgery is a common phenomenon in PTCs and MTCs as well. Of course, some of these tumors show a spontaneous aggressive change or an acceleration in their growth rate [17]. Acceleration in the tumor growth rate is typically seen in cases of poor differentiation and anaplastic change of differentiated thyroid carcinoma.

Our study has several limitations. The study design was retrospective, and the number of patients was small (*n* = 46) because we used only the data of a new Ct measurement method by ECLIA after April 2015. Second, in this thought-experiment study, we compared the Ct-DR with a hypothetical TV-DR. Although both values would reflect the tumor growth rate, the bases of their calculations are different. Since active surveillance for MTCs was not performed (unlike papillary thyroid microcarcinomas), our patients’ TV-DR values after surgery based on the actual tumor size measurement were not available. However, our present findings provide some insight into the natural history of MTCs. Third, the number of hereditary patients was small at 18 and it was not possible to analyze according to *RET* mutations.

## Conclusions

In the presented 46 patients with MTCs, the calculated hTV-DRs before surgery were significantly higher than the observed Ct-DRs after surgery, suggesting that there were rapid growth periods before surgery in the vast majority of the patients.

## Data Availability

All data is contained within the manuscript and additional files.

## Abbreviations

MTC: Medullary thyroid carcinoma
Ct: Calcitonin
DT: Doubling time
TV: Tumor volume
DR: Doubling rate
ATA: American Thyroid Association
ECLIA: Electrochemiluminescence immunoassay
CEA: Carcinoembryonic antigen
PTC: Papillary thyroid carcinoma
